# Safety and immunogenicity of a modified Omicron-adapted inactivated vaccine in healthy adults: a randomized, double-blind, active-controlled Phase III clinical trial

**DOI:** 10.3389/fimmu.2023.1241153

**Published:** 2023-09-18

**Authors:** Jialei Hu, Yueyue Liu, Shuo Liu, Qun Shu, Xuenan Yang, Kai Chu, Yaping Qiao, Yaling Hu, Kaiqin Wang, Hongxing Pan

**Affiliations:** ^1^ Department of Vaccine Clinical Evaluation, Jiangsu Provincial Center for Disease Control and Prevention, Nanjing, Jiangsu, China; ^2^ Division of Respiratory Virus Vaccines, Institute for Biological Product Control, National Institutes for Food and Drug Control, Beijing, China; ^3^ Clinical Research and Development Center, Sinovac Biotech Co., Ltd., Beijing, China; ^4^ Statistics and Decision Science, Beijing Key Tech Statistics Technology Co., Ltd., Beijing, China; ^5^ Center of Research and Development, Sinovac Life Sciences Co., Ltd., Beijing, China

**Keywords:** SARS-CoV-2, Omicron-adapted vaccine, immune responses, safety, adults

## Abstract

**Background:**

Updated vaccine strategies are needed to protect against new SARS-CoV-2 variants with increased immune escape. Here, information on the safety and immunogenicity of an inactivated Omicron-adapted vaccine is presented, as compared with CoronaVac.

**Methods:**

A randomized, double-blind, active-controlled, phase III clinical trial was conducted to compare a modified Omicron-adapted vaccine (Omicron vaccine) with the authorized prototype vaccine (CoronaVac®) as a booster dose. Healthy adults aged ≥18 years, who have previously received 2 or 3 doses of CoronaVac (2C or 3C cohort) at least 6 months before, were enrolled to get a booster dose of Omicron vaccine or CoronaVac in a ratio of 2:1 (2C/3C+1O/1C). Back-up serums after two initial doses of CoronaVac (2C+0) for adults aged 26-45 years were collected from a previous study. Immunogenicity and safety data at 28 days after vaccination were collected and analyzed. One of the primary objectives was to evaluate the superiority of immunogenicity of Omicron vaccine booster against Omicron BA.1, compared with CoronaVac booster against BA.1. Another objective was to evaluate the non-inferiority of immunogenicity of Omicron vaccine booster against BA.1, compared with two initial doses of CoronaVac against ancestral strain.

**Results:**

Between June 1^st^ and July 21^st^, 2022, a total of 1,500 healthy adults were enrolled. Results show that all pre-specified superiority criteria for BA.1 neutralizing antibody were met. Specifically, within the 3C cohort (3C+1O vs. 3C+1C), the geometric mean titers’ (GMT) ratio and 95% confidence interval (CI) was 1.64 (1.42, 1.89), with the lower 95%CI ≥1; a GMT ratio of 1.84 (1.57, 2.16) was observed for 2C+1O versus 3C+1C. For seroconversion rate, the lower 95%CIs of differences between immuno-comparative groups (2/3C+1O vs. 3C+1C) were all above the superiority criterion 0%. However, the non-inferiority criterion of the lower 95%CI of GMT ratio ≥2/3 was unfulfilled for 2C/3C+1O against BA.1 versus 2C+0 against ancestral strain. Safety profiles were similar between groups, with no safety concerns identified.

**Conclusion:**

The Omicron-adapted vaccine was well-tolerated and could elicit superior immune responses as compared with CoronaVac against Omicron, while it appeared inferior to CoronaVac against ancestral strain.

**Clinical trial registration:**

https://classic.clinicaltrials.gov/ct2/show/NCT05381350?term=NCT05381350&draw=2&rank=1, identifier NCT05381350.

## 1 Introduction

The emergence of severe acute respiratory syndrome coronavirus 2 (SARS-CoV-2) in 2019 has caused extraordinary threats to human health. During the pandemic, SARS-CoV-2 has evolved into five variants of concern (VOCs), including Omicron the most antigenically divergent variant so far (1). The Omicron BA.1 variant (B.1.529), which contains more than 30 amino acid mutations in the spike protein, is firstly detected in South Africa in November 2021, and then rapidly spreads around the world (2). Since its emergence, Omicron and its subvariants (e.g., BA.2, BA.4, BA.5, BF.7, XBB) have been the key driver of the pandemic, resulting in several new waves of infection (3).

To fight against the challenge of coronavirus disease 2019 (COVID-19) pandemic, various types of technologies have been applied concurrently in the development of COVID-19 vaccines, including inactivated, protein subunit, viral vector, or RNA-based platforms (4). Inactivated vaccine, containing the whole viral antigen of SARS-CoV-2 ancestral strain, showed favorable efficacy or effectiveness with regard to preventing COVID-19, and played a unique role in elevating vaccine availability worldwide in the early stages of the pandemic. CoronaVac manufactured by Sinovac Life Science Co., Ltd. has been validated by the World Health Organization (WHO) for emergency use and been authorized by more than 60 countries and jurisdictions (5). In a large phase III trial in Turkey, two-dose CoronaVac showed an acceptable safety profile and an efficacy of 83.5% against symptomatic COVID-19 (6). Consistent real-word evidence has also demonstrated the effectiveness of CoronaVac for preventing COVID-19 (7). However, circulating Omicron-associated variants have imposed great pressure on vaccine-derived immunity due to their substantial transmissibility and immune evasion properties (8, 9), although booster doses could provide substantial additional protection against severe disease and death (10, 11). With the expectation that SARS-CoV-2 would continue to evolve over time, modified or next-generation COVID-19 vaccines with enhanced immune responses were needed.

In accordance with the guidance of WHO and Food and Drug Administration (FDA), a modified vaccine refers to a vaccine against a variant for which the mere change is the virus strain in an authorized prototype vaccine, aiming to improve the ability against SARS-CoV-2 variants (12, 13). The guidelines also consider an alternative immuno-bridging trial to compare immune responses induced by the modified vaccine and the authorized vaccine. Currently, bivalent mRNA vaccines (ancestral strain and Omicron BA.1, and ancestral strain and Omicron BA.4/BA.5) showed superior neutralizing antibody responses against Omicron variants as compared with the prototype mRNA vaccines (14–17), and have been authorized as boosters in the United States and/or elsewhere (18–20).

Sinovac has also developed an Omicron-adapted inactivated vaccine (Omicron vaccine) following the same manufacturing process as CoronaVac but containing Omicron BA.1 instead in early 2022. Here, we summarize the safety and immunogenicity results of this Omicron vaccine used as a booster dose from a phase III immuno-bridging clinical trial.

## 2 Materials and methods

### 2.1 Study design and participants

A single-center, randomized, double-blind, active-controlled, phase III immuno-bridging clinical trial was conducted, aiming to assess the immunogenicity and safety of Omicron vaccine as compared with the authorized prototype vaccine CoronaVac in Jiangsu Province, China. Healthy adults aged 18 years and older, without a history of or current SARS-CoV-2 infection, were eligible for enrolment. Participants should have previously received either 2 or 3 doses of CoronaVac at least 6 months before (2C or 3C cohorts), for which the time intervals were 21-60 days between the first and second doses and at least 6 months between the second and third doses. Within each cohort, enrolled eligible participants were randomly assigned, in a ratio of 2:1, to receive one booster dose of Omicron vaccine (the trial group) or CoronaVac (the control group), and the first 80 participants in the trial group and the first 40 participants in the control group were assigned to the immunogenicity subgroups. Randomization codes were generated by the statistician using stratified block randomization, according to age stratification (18 to 59 years, or ≥60 years; 4:1 ratio). The randomization code was allocated to each participant in the sequence of enrolment order, and then the participants received the investigational products labeled with the same code. Concealed random group allocations and blinding codes were kept in sealed envelopes, and investigators, participants, and laboratory staff were all blinded to the group allocation. Detailed information on the inclusion/exclusion criteria for participants is listed in the Appendix.

This trial was approved by the ethics committee of Jiangsu Provincial Centre for Disease Control and Prevention. Written informed consent was obtained from all participants before screening, and the trial was conducted in accordance with the principles of the International Council for Harmonisation of Technical Requirements for Registration of Pharmaceuticals for Human Use, the standards of Good Clinical Practice, and Chinese regulatory requirements. The trial was registered in the ClinicalTrials.gov registration database with an identifier of NCT05381350.

### 2.2 Study vaccines

Except for the SARS-CoV-2 antigen, Omicron vaccine was following the same manufacturing process and facilities for vaccine production of the licensed prototype vaccine CoronaVac as has been described previously (21–23). In brief, the inactivated Omicron and CoronaVac vaccines were created from African green monkey kidney cells (Vero cells), which had been inoculated with SARS-CoV-2 Omicron BA.1 strain or ancestral strain, respectively. The two SARS-CoV-2 strains were then harvested, inactivated with β-propiolactone, concentrated, purified, and finally adsorbed onto aluminum hydroxide. Both vaccines were prepared in a Good Manufacturing Practice-accredited facility of Sinovac, which was periodically inspected by the National Medical Products Administration (NMPA) committee for compliance. Vaccines were prefilled in ready-to-use syringes and were designed to be administered intramuscularly at a dose of 1200SOU/0.5ml (6μg antigen) for Omicron vaccine and 600SU/0.5ml (3μg antigen) for CoronaVac. In this trial, the schedules of both vaccines were one booster dose for all participants.

### 2.3 Safety assessment

The safety objectives were to evaluate the safety and reactogenicity of one booster dose of Omicron vaccine as compared with CoronaVac. Participants were observed in the trial center for at least 30 min after vaccination. For the first 7 days, participants were required to record any local adverse events at the injection site and systemic adverse events on diary cards. Solicited local adverse events included injection site pain, induration, swelling, erythema, rash, and pruritus. Solicited systemic adverse events included fever, acute hypersensitive reaction, diarrhea, nausea, myalgia, headache, cough, and fatigue. Investigators then conducted a face-to-face interview to confirm safety profiles on Day 7. From Day 8 to 28, adverse events were collected by spontaneous records or reports from participants combined with regular visits on Day 14 (only for immunogenicity subgroup participants) and Day 28. Serious adverse events (SAE) and adverse events of special interest (AESI) were recorded throughout the trial until 12 months after vaccination. The reported adverse events were graded according to the NMPA guidelines (24). The causal relationship between adverse events and vaccination was established by investigators.

Safety assessments in this article included solicited local and systemic adverse reactions and unsolicited adverse reactions, as well as SAE and AESI within 28 days after vaccination. The follow-up for SAE and AESI is continuing.

### 2.4 Immunogenicity assessment

For all participants, serum samples were collected on the day of booster dose (before vaccination) and at 28 days after vaccination. For immunogenicity subgroup participants, extra blood samples at 7 and 14 days after vaccination were collected to explore whether booster vaccination could induce earlier immune response. Previous back-up serum samples at 28 days after two initial doses of CoronaVac were also collected (the historical control group), in adults aged 26-45 years old from the previous lot-to-lot consistency study (25). Neutralizing antibody titers against ancestral strain, Delta, and Omicron BA.1 and BA.5 were then tested using a micro cytopathogenic effect assay, which was done by the National Institute for Food and Drug Control (NIFDC). Details on immunological assessment methods and related testing procedures are described in the Appendix.

There were two primary objectives. One of them was to evaluate the superiority of neutralizing antibody response against Omicron BA.1 at 28 days after Omicron vaccine booster as compared with CoronaVac booster, based on the geometric mean titers’ (GMT) ratio and on the seroconversion rate difference. It is worth noting that the 3C control group was used for either 2C or 3C trial group according to the requirement of NMPA (2/3C+1O vs. 3C+1C). The other one was to evaluate the non-inferiority of neutralizing antibody response against Omicron BA.1 at 28 days after one booster dose of Omicron vaccine, compared with the immune response against ancestral strain at 28 days after two initial doses of CoronaVac, based on GMT ratio (2/3C+1O vs. 2C+0).

### 2.5 Statistical analysis

The statistical analyses were done separately for two different cohorts (2C and 3C cohorts). Safety profiles were assessed in the safety set (SS) of participants who received one booster dose. Immunological endpoints were assessed in the per-protocol set (PPS), which included all participants who completed one booster dose and had available neutralizing antibody results according to the protocol, as well as all back-up serum samples with available neutralizing antibody results. According to the timepoints postvaccination, PPS was divided into PPS1 (Day 7), PPS2 (Day 14) and PPS3 (Day 28). The demographics of participants in the full analysis set (FAS) were summarized by cohorts and vaccination groups. Pearson chi-squared test or Fisher’s exact test were applied to analyze categorical variables for group comparison, and t-tests or analysis of variance (ANOVA) were used for continuous variables.

For the primary objectives, superiority is considered to be fulfilled when the lower boundary of 95% confidence intervals (CIs) for the GMT ratio is >1, which is equivalent to the GMT difference after log_10_ transformation greater than 0, and the lower boundary of 95%CI for the seroconversion rate difference is >0%. Noninferiority is considered to be fulfilled when the lower boundary of 95%CI for the GMT ratio is ≥2/3, which is equivalent to the GMT difference after log_10_ transformation at least -0.176. The immunogenicity was assessed in 1,500 enrolled participants, of which 750 participants were in each cohort (500 and 250 in the trial and control groups, respectively), as well as 250 back-up serum samples in the historical control group. This sample size would allow for a statistical power of 92% to detect superiority in terms of an expected GMT difference of 0.2 (on a log_10_ scale, with a standard deviation [SD] of 0.55) and a statistical power of 98% to detect superiority in terms of an expected seroconversion rate difference of 20%, with an expected drop-out rate of 20% and a two-sided significance level of 5%. This sample size would also allow for a statistical power of 90% to detect noninferiority in terms of an expected GMT difference of 0 (on a log_10_ scale, with a SD of 0.60), with an expected drop-out rate of 20% and a two-sided significance level of 5%. The sample size was calculated using PASS 2022.

Observed GMTs and corresponding 95%CIs were calculated based on a standard normal distribution of log-transformed neutralizing antibody titers, of which titers lower than the lower limit of quantitation (LLOQ, 1:4) were presented as half of LLOQ. Analysis of covariance (ANCOVA) was used to compare the difference between groups, which used log-transformed antibody titers after vaccination as the dependent variable, study vaccines and age (18-59 or ≥60 years) as the fixed effects, and log-transformed antibody titers before vaccination as the covariate. GMTs and 95% CIs were then estimated by the geometric least-square mean from the ANCOVA model, and the differences in antibody responses between groups were estimated by the ratio of geometric least-square mean and 95% CIs. Seroconversion refers to GMT changes from <LLOQ to ≥4-fold LLOQ (1:16) after vaccination, or at least 4-fold increases after vaccination if baseline GMT is ≥LLOQ. The corresponding 95%CIs of seroconversions were calculated according to Clopper-Pearson analysis, and seroconversion rate differences and 95%CIs between groups were estimated by the age stratified (18-59 or ≥60 years) Cochran-Mantel-Haenszel test.

Subgroup analyses were performed by age (18-59 and ≥60 years) for neutralizing antibodies. Exploratory analyses were applied to evaluate the antibodies response at 7 and 14 days after vaccination in the immunogenicity subgroup participants. Hypothesis testing was two-sided, and p values of less than 0.05 were considered to be significant. All analyses were conducted with SAS (Version 9.4, SAS Institute Inc., Cary, USA).

## 3 Results

### 3.1 Participant characteristics

**Figure 1:**
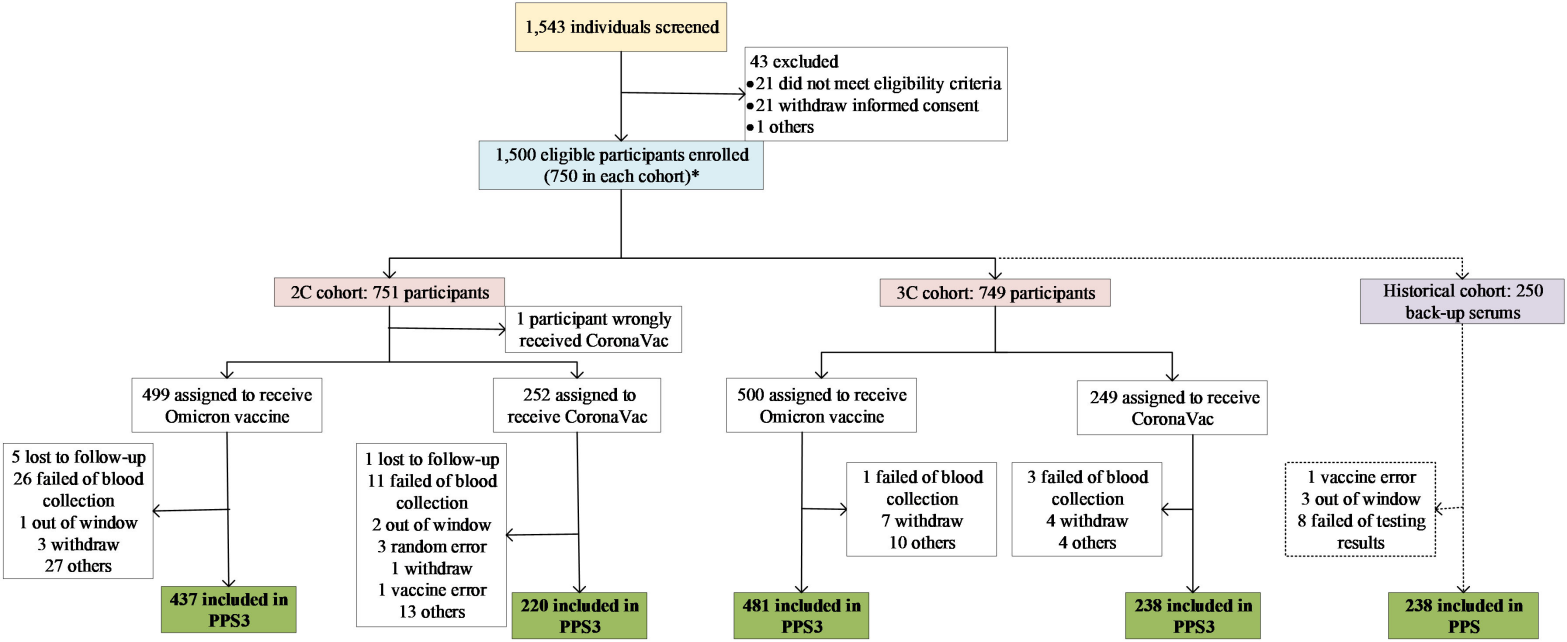
Study profile. *: One participant should be in 2C cohort but was wrongly allocated into 3C cohort in randomization procedure. In the statistical analysis, the participant was swich to 2C cohort.

Between June 1^st^ and July 21^st^, 2022, a total of 1500 eligible participants were enrolled (**Figure 1**). Given that one participant was wrongly distributed, 751 (499 and 252) and 749 (500 and 249) participants were finally allocated into 2C and 3C cohorts to get vaccine, respectively, and were included in the SSs. Among these participants who were vaccinated, some of them were not included in the PPSs, due to failed of blood sampling, lost to follow-up, out of window visit, and so on. In 2C cohort, 657 out of 751 participants (87.5%; 437 and 220) were included in the PPS3, and 114 (95.0%; 78 and 36) participants were in the PPS1 and PPS2. In 3C cohort, 730 out of 749 participants (97.5%; 481 and 249) were included in the PPS3, and 114 (95.0%; 76 and 38) and 115 (95.8%; 77 and 38) participants were in the PPS1 and PPS2, respectively. In addition, 250 serum samples were selected from the previous lot-to-lot trial, of which 238 (95.2%) were included in the PPS (**Figure 1**).

The baseline characteristics of participants were similar between the trial and control groups within 2C or 3C cohort, respectively (**Table 1**). In 2C cohort, the average ages were 43.5 years in the trial group and 42.7 years in the control group, and 60.44% and 66.14% were male, respectively; the time intervals to the latest dose were similar between groups, with median values of 342 days (range from 183 to 407) and 349 days (208 to 402), respectively. Comparative baseline characteristics were also observed within 3C cohort. Among participants aged 18-59 or ≥60 years, similar baseline characteristics were also observed between groups within each cohort, as shown in **Supplementary Table 1**.

**Table 1:** Baseline demographic and clinical characteristics of participants.

Characteristic	2C cohort		3C cohort		P value*	Historical cohort
	Omicron vaccine booster (N=498)	CoronaVac booster (N=251)	Omicron vaccine booster (N=499)	CoronaVac booster (N=249)		(N=250)
Age, years						
Mean (SD)	43.5 (14.5)	42.7 (14.6)	48.9 (12.0)	49.0 (11.8)	<0.0001	34.8 (5.4)
Median (range)	39 (18, 77)	39 (18, 74)	50 (21, 77)	51 (20, 73)		34 (26, 45)
Age subgroup, n (%)						
18-59 years	399 (80.12)	203 (80.88)	396 (79.36)	199 (79.92)	0.9484	250 (100.00)
≥60 years	99 (19.88)	48 (19.12)	103 (20.64)	50 (20.08)		0 (0.00)
Male, n (%)	301 (60.44)	166 (66.14)	231 (46.29)	129 (51.81)	0.0244	118 (47.20)
Ethnic, n (%)						
Han	498 (100.00)	251 (100.00)	498 (99.80)	249 (100.00)	–	249 (99.60)
Hui	0 (0.00)	0 (0.00)	1 (0.20)	0 (0.00)		1 (0.40)
Height, m						
Mean (SD)	1.66 (0.09)	1.68 (0.08)	1.64 (0.08)	1.64 (0.08)	0.0029	1.66 (0.08)
Weight, kg						
Mean (SD)	70.5 (13.8)	73.8 (15.2)	68.8 (12.4)	69.7 (13.2)	0.4781	71.5 (14.8)
Time to the latest dose, days						
Mean (SD)	338.3 (32.5)	341.1 (29.4)	203.2 (14.7)	202.7 (14.6)	<0.0001	NA
Median (range)	342 (183, 407)	349 (208, 402)	204 (167, 241)	204 (181, 235)		NA

Results are shown for the FAS population. *: P values were calculated for comparison between the 2C trial group and the 3C control group.

Given that the 2C and 3C trial groups shared the same control group (the 3C control group) in the primary immunogenicity analysis, participants’ demographic characteristics across cohorts were also shown (**Table 1**). There were significant differences in age (p<0.0001) and gender (p=0.0244) distributions between the 2C trial group and the 3C control group, and a longer time interval to the latest dose was observed in the 2C trial group (median: 342 vs. 204 days; p<0.0001). Compared with the historical control group, the average ages in the 2C and 3C trial groups were higher (43.5 and 48.9 vs. 34.8 years).

### 3.2 Safety

A total of 183 out of 1499 participants (12.21%) reported adverse reactions within 28 days after booster dose (**Table 2** and **Supplementary Table 2**). Occurrences of adverse reactions were similar between the trial (114 out of 998 participants, 11.42%) and control groups (69 out of 501 participants, 13.77%), and the majority of them were solicited (proportions of 97.37% and 100%, respectively). The incidences of solicited local adverse reactions were 6.71% for Omicron vaccine and 9.38% for CoronaVac, of which injection-site pain was the most reported event (5.61% and 7.98%), followed by induration (1.20% and 2.59%) and pruritus (1.00% and 2.40%). The most frequent solicited systemic adverse reactions were fever, with incidence rates of 3.51% and 4.39%, respectively. Unsolicited adverse reactions occurred in 3 participants (0.30%) in the trial group and 1 participant (0.20%) in the control group. Most of adverse reactions were mild (grades 1) for both vaccine boosters in two groups, with proportions of 92.81% and 97.46%. Two adverse reactions in grade 3 occurred, which were solicited events of fever and injection-site redness in the trial group. No grade 4 events, SAEs and AESIs related to vaccines were observed.

**Table 2:** Solicited (local and systemic) and unsolicited adverse reactions (AR) within 28 days after booster doses, according to Grade.

2C and 3C combined cohort	All participants		
	Omicron vaccine booster group (N=998)	CoronaVac booster group (N=501)	P value
	n (%)	n (%)	
**Total AR**	114 (11.42)	69 (13.77)	0.2096
Grade 1	107 (10.72)	69 (13.77)	0.0891
Grade 2	9 (0.90)	2 (0.40)	0.3538
Grade 3	2 (0.20)	0 (0.00)	0.5547
**Solicited AR**	111 (11.12)	69 (13.77)	0.152
**Local AR**	67 (6.71)	47 (9.38)	0.0787
Pain	56 (5.61)	40 (7.98)	0.0929
Induration	12 (1.20)	13 (2.59)	0.055
Pruritus	10 (1.00)	12 (2.40)	0.0411
Erythema	7 (0.70)	6 (1.20)	0.3789
Swelling	5 (0.50)	8 (1.60)	0.0395
Rash	2 (0.20)	0 (0.00)	0.5547
**Systemic AR**	58 (5.81)	29 (5.79)	1
Fever	35 (3.51)	22 (4.39)	0.3939
Diarrhea	10 (1.00)	3 (0.60)	0.5617
Fatigue	9 (0.90)	4 (0.80)	1
Headache	6 (0.60)	6 (1.20)	0.2312
Myalgia	4 (0.40)	1 (0.20)	0.6698
Cough	3 (0.30)	1 (0.20)	1
Acute hypersensitive reaction	2 (0.20)	0 (0.00)	0.5547
Nausea	1 (0.10)	0 (0.00)	1
**Unsolicited AR**	3 (0.30)	1 (0.20)	1
Oropharynx pain	1 (0.10)	0 (0.00)	1
Constipation	1 (0.10)	0 (0.00)	1
Dizzy	1 (0.10)	0 (0.00)	1
Vesicular exanthema	0 (0.00)	1 (0.20)	0.3342

For the age subgroups of 18-59 and ≥60 years, the overall incidence rates of adverse reactions within 28 days were 12.68% (152 out of 1199 participants) and 10.33% (31 out of 300 participants), respectively, with no differences between the trial and control groups (**Supplementary Table 2**). For the trial group with Omicron vaccine, the frequency of adverse reactions was 7.43% in participants aged ≥60 years, which was relatively lower than that in participants aged 18-59 years (12.44%). In addition, no unsolicited adverse reactions and grade 3 adverse reactions were observed for participants aged ≥60 years.

### 3.3 Immunogenicity

The immunogenicity results are shown in **Table 3** and **Supplementary Table 3**. In participants within 3C cohort, the observed GMTs and 95%CIs of BA.1 neutralizing antibody at 28 days after vaccination were 10.36 (9.39, 11.44) for Omicron vaccine and 6.52 (5.73, 7.43) for CoronaVac (**Table 3**). The estimated GMTs were 9.53 (8.65, 10.50) and 5.81 (5.11, 6.61), respectively, resulting in a GMT ratio of 1.64 (1.42, 1.89). Similarly, a GMT ratio of 1.84 (1.57, 2.16) was observed when comparing the estimated GMT in the 2C trial group [8.87 (7.98, 9.87)] with that in the 3C control group [4.81 (4.20, 5.52)]. In addition, the superiority criteria for seroconversion rate differences between immuno-comparative groups were also fulfilled, which were 17.73% (11.46, 24.00) between the 3C trial and control groups, and 19.04% (12.62, 25.45) between the 2C trial group and the 3C control group. In subgroup analyses by age, as shown in **Supplementary Table 3**, the superiority criteria were all met for participants either aged 18-59 or ≥60 years, except for the seroconversion rate difference between the 2C trial group and the 3C control group in participants aged ≥60 years [9.13% (-4.72, 20.92)].

**Table 3:** Primary immunogenicity analysis on Day 28 after booster doses of Omicron vaccine and CoronaVac, as well as after two initial doses of CoronaVac.

Variable	Omicron vaccine booster		CoronaVac booster	Historical control group
	2C cohort	3C cohort	3C cohort	
**N**	**437**	**481**	**238**	**238**
**Against SARS-CoV-2**	**Omicron BA.1**	**Omicron BA.1**	**Omicron BA.1**	**ancestral strain**
Day 0				
Observed GMT (95%CI)	2.02 (2.00, 2.05)	2.65 (2.52, 2.78)	2.74 (2.52, 2.97)	/
Day 28				
Observed GMT (95%CI)	9.30 (8.45, 10.24)	10.36 (9.39, 11.44)	6.52 (5.73, 7.43)	45.97 (41.08, 51.44)
GMI (95%CI)	4.60 (4.18, 5.06)	3.91 (3.59, 4.27)	2.38 (2.14, 2.66)	/
Seropositive rate (95%CI)	59.04% (54.27, 63.69)	63.83% (59.35, 68.13)	44.54% (38.12, 51.10)	97.48% (94.59, 99.07)
Superiority test				
Estimated GMT (95%CI)	8.87 (7.98, 9.87)	9.53 (8.65, 10.50)	4.81 (4.20, 5.52)# 5.81 (5.11, 6.61)¶	/
**GMT ratio (95%CI)**	**1.84 (1.57, 2.16)**	**1.64 (1.42, 1.89)**	Ref	/
Seroconversion rate (95%CI)	35.01% (30.54, 39.69)	33.68% (29.46, 38.10)	15.97% (11.55, 21.25)	/
**Seroconversion rate differences (95%CI)**	**19.04% (12.62, 25.45)**	**17.73% (11.46, 24.00)**	Ref	/
Non-inferiority test				
Estimated GMT (95%CI)	9.30 (8.48, 10.19)	10.36(9.45, 11.37)	/	45.97(40.60, 52.04)# 45.97(40.28, 52.45)¶
**GMT ratio (95%CI)**	**0.20 (0.17, 0.24)**	**0.23 (0.19, 0.26)**	/	Ref

Results are shown for the participants in PPS3. #: Estimated GMT value when comparing with Omicron vaccine group in 2C cohort. ¶: Estimated GMT value when comparing with Omicron vaccine group in 3C cohort. The positive cutoff value of the neutralizing antibody titer was 1:8. GMI, geometric mean increase. The bold values indicate the results of primary endpoints in this study.

Comparing with the historical control group, not all pre-specified non-inferiority criteria were met (**Table 3**). Specifically, the estimated GMT ratios for the 2C and 3C trial groups against Omicron BA.1 as compared with the historical control group against ancestral strain were 0.20 (0.17, 0.24) and 0.23 (0.19, 0.26), respectively. When restricting to participants aged 18-59 years, the non-inferiority criteria were still not fulfilled, with GMT ratios of 0.22 (0.19, 0.26) and 0.24 (0.20, 0.28), respectively (**Supplementary Table 3**).

**Figure 2:**
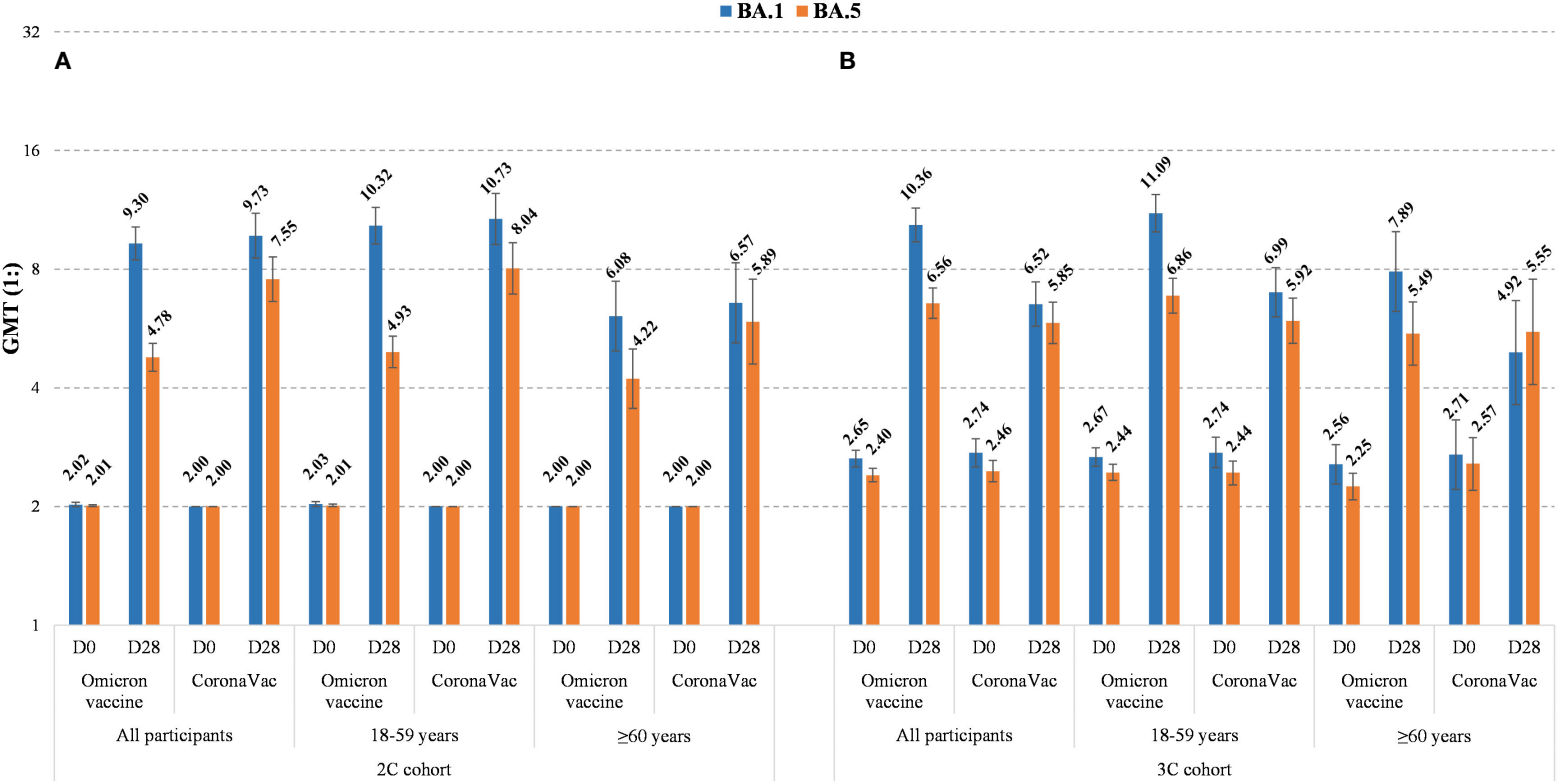
Observed neutralizing antibody levels against Omicron BA.1 and BA.5 before and 28 days after booster doses of Omicron vaccine or CoronaVac in 2C cohort **(A)** and 3C cohort **(B)**, by age (all, 18-59 years, and ≥60 years). The GMTs are shown on the top of histograms and their 95% CIs are indicated by the error bars.

Detailed observed neutralizing antibody levels against Omicron BA.1, BA.5, Delta and ancestral strains are shown in **Figure 2** and **Supplementary Figure 1**. Pre-vaccination GMTs in 3C cohort were higher than those in 2C cohort for all strains in all age groups. For the trial groups 28 days after vaccination, GMTs against Omicron BA.1 in 3C cohort were slightly higher than those in 2C cohort, whereas for the control groups GMTs against ancestral strain in 3C cohort were lower than those in 2C cohort in all age groups. Additionally, in either 2C or 3C cohort 28 days after vaccination, GMTs against Omicron BA.5 were relatively lower than those against Omicron BA.1, and GMTs against ancestral strain were much higher than those against Omicron BA.5 and BA.1. The neutralizing antibody levels at 7 and 14 days after vaccination are shown in **Supplementary Figure 2** and **Supplementary Table 4**. Results showed that GMTs for all strains appeared to sharply increase at the first 7 days and then reached the highest levels at 14 days.

## 4 Discussion

In this trial, the booster dose of Omicron-adapted vaccine elicited superior neutralizing antibody responses against Omicron BA.1 compared to CoronaVac booster, which was demonstrated by the immunogenicity endpoints of GMT ratio and seroconversion rate difference following the guidelines of regulatory authorities. These results were comparable to other Omicron vaccines. Pfizer-BioNTech and Moderna have each developed two Omicron-containing vaccines, which are bivalent mRNA vaccines of ancestral and Omicron BA.1 as well as ancestral and Omicron BA.4/BA.5. In their clinical trials, the second booster dose of bivalent vaccines was administrated to participants who had received three prior doses of prototype vaccines (14–17). They observed that the neutralizing antibody titers against Omicron variants induced by bivalent vaccines were superior to the prototype vaccines. Specifically, for ancestral/Omicron BA.1 bivalent vaccines of Pfizer-BioNTech and Moderna, the GMT ratios against Omicron BA.1 28 days after vaccination were 1.56 (1.17, 2.08) and 1.75 (1.49, 2.04), and the seroconversion rate differences were 14.60% (4.00, 24.90) and 1.50% (-1.10, 4.00), respectively (15, 16). A monovalent Omicron BA.1 vaccine, developed by Pfizer-BioNTech, also showed superior immunogenicity against Omicron BA.1, with GMT ratio and seroconversion rate difference of 2.23 (1.65, 3.00) and 19.60% (9.30, 29.70), respectively (16). Overall, these consistent results implied that Omicron-adapted vaccines can induce better immune responses against variants as compared with the prototype vaccines, emphasizing the use of Omicron-adapted vaccines to maximize protection.

The setting of a non-inferiority criterion based on humoral immune response levels could be an alternative approach to extrapolate the efficacy of Omicron vaccine as compared with the immune response of two initial doses of CoronaVac, for which the efficacy had been previously documented (26). However, our study did not fulfill the pre-specified non-inferiority criterion. This finding might be explained by the weak and limited natural immunity of Omicron compared with ancestral strains (27), and could also be interpreted by a process called ‘immune imprinting’ (somewhat related to the ‘original antigenic sin’ concept) (28), which is that previous infection/vaccination are imprinted on the immune system and can impair the ability of boosting immunity against subsequent variants. Immune imprinting effect was also shown in a study on 731 UK healthcare workers who received three doses of mRNA vaccine and were infected during Omicron wave (29). Further studies are needed to explore the mechanism of immunogenicity, as well as whether the cellular immune response after Omicron vaccine booster shows a similar phenomenon as well.

Additionally, in our study, the antibody response against Omicron BA.1 after Omicron vaccine booster was much weaker than that against ancestral strain, with GMTs of 9.30 vs. 56.51 and 10.36 vs. 84.41 in 2C and 3C cohorts, respectively. Similarly, for Pfizer-BioNTech’s monovalent BA.1 vaccine booster, the GMT against Omicron BA.1 and ancestral strain was 1015 and 5539, respectively (15). These results imply that the immune system of people who had previously been vaccinated was primed to respond to ancestral strain rather than Omicron variant after getting a booster dose, which is also related to the immune imprinting theory.

Another concern is that Omicron is mutating further, perhaps leading to easier breaking of current vaccine protection. In our study, we observed that Omicron vaccine booster induced much lower neutralizing antibody titers to Omicron BA.5 as compared with against Omicron BA.1. Similar trends were also found in Pfizer-BioNTech and Moderna’s bivalent (ancestral and Omicron BA.1) booster vaccines, of which the GMTs against Omicron BA.4/5 and BA.1 were 114 vs. 711 and 727 vs. 2372, respectively (15, 16). For Moderna’s bivalent (ancestral and Omicron BA.4/5) booster vaccine, the GMTs against BQ1.1 and XBB.1 were much lower than that against Omicron BA.4/5 after booster vaccination (14). These findings underscore the importance of real-world evidence on the effectiveness of authorized bivalent mRNA vaccines on Omicron BQ.1.1, XBB.1, and new emerging sublineages.

Breaking immune imprinting has become the key issue for new variant vaccine development. Given the waning humoral and cellular immune responses after prior infection or vaccination in previous months (30, 31), there is a possibility that the immune system may forget prior vaccination/infection after enough time passed. Our results showed higher antibody levels in the 2C control groups as compared with the 3C control group, which implied that time interval to the latest dose, rather than the number of prior doses, might play a vital role in immune response to current vaccines. Therefore, keeping booster doses relatively spaced out may give the immune system more time to mount a more effective response to new variants, but this will need future studies.

Limitations of this trial included that only humoral immune response against Omicron variants was assessed, whereas cellular immune response also contributes to protection against infection and severe disease (32). Real-world studies have demonstrated that booster vaccination of CoronaVac provided substantial protection against severe COVID-19 during Omicron period (10, 33), which could be attributable to robust cellular immunity. Future work is therefore needed to evaluate the cellular immunity of Omicron vaccine. Furthermore, given the objective situation of vaccination campaigns in China, the temporal intervals to the latest doses were different between 2C and 3C cohorts and cannot be controlled by sensitive analysis as well. Finally, we reported the results at 28 days after booster doses but long-term follow-up is still ongoing. Further evaluation on immunity persistence is needed.

In conclusion, a booster dose of Omicron-adapted vaccine is well-tolerated and can elicit a superior immune response against Omicron BA.1 as compared with CoronaVac; however, the humoral immune response induced is weak.

## Data availability statement

The datasets presented in this article are not readily available because the original contributions presented in the study are included in the article/**Supplementary Material**. Further inquiries can be directed to the corresponding authors. Requests to access the datasets should be directed to HP, panhongxing@126.com.

## Ethics statement

The studies involving humans were approved by the ethics committee of Jiangsu Provincial Center for Disease Control and Prevention, China. The studies were conducted in accordance with the local legislation and institutional requirements. The participants provided their written informed consent to participate in this study.

## Author contributions

JH, YL, and SL participated in manuscript drafting. SL, KC, YQ, YH, and HP contributed to study conception and design. QS contributed to statistical analysis. JH, XY, KC, and HP recruited participants and conducted the study. YL and KW conducted sample testing. YH, KW, and HP revised the manuscript. All authors contributed to the article and approved the submitted version.
