# Supplementary material for: Safety and immunogenicity of a modified Omicron-adapted inactivated vaccine in healthy adults: a randomized, double-blind, active-controlled Phase III clinical trial
Source: Front Immunol. 2023 Sep 18;14:1241153. doi: 10.3389/fimmu.2023.1241153 (PMC10548824; doi:10.3389/fimmu.2023.1241153)
Supplement: Supplementary file 1 [file DataSheet_1.docx]

Supplementary Material

**Jialei Hu ^1†^, Yueyue Liu ^2†^, Shuo Liu ^3†^, Qun Shu ^4^, Xuenan Yang ^3^, Kai Chu ^1^, Yaping Qiao ^3^, Yaling Hu ^5*^, Kaiqin Wang ^2*^, Hongxing Pan ^1*^**

^†^These authors (Jialei Hu, Yueyue Liu, Shuo Liu) contributed equally to this work and share first authorship.

**^*^Correspondence:** Yaling Hu: huyl@sinovac.com; Kaiqin Wang: ahaqincaicai@163.com; Hongxing Pan: panhongxing@126.com.

**1. Study Eligibility Criteria**

**1.1 Inclusion Criteria**

1. Healthy adults aged 18 years and above.
2. Participants who have completed 2 doses of CoronaVac or 3 doses of CoronaVac at least 6 months before (the interval between the first dose and the second dose was 21-60 days, and the interval between the second dose and the third dose was 6 months or more).
3. Participants can understand and voluntarily sign the written informed consent.
4. Provide legal identification and vaccination certificate of CoronaVac.

**1.2 Exclusion Criteria**

1. History of SARS-CoV-2 infection (laboratory confirmed).
2. Close contact with a confirmed COVID-19 cases (nucleic acid PCR test or antigen test positive patients) within 14 days prior to enrollment.
3. Received other types of COVID-19 vaccine previously except for two or three doses of CoronaVac.
4. Allergic to vaccines or vaccine ingredients, serious adverse reactions to vaccines, such as urticaria, dyspnea, angioneurotic edema.
5. With autoimmune disease and/or blood disease history (including but not limited to systemic lupus erythematosus, thyroidectomy, autoimmune thyroid disease, any form of malignancy, absence of spleen, functional absence of spleen, or splenectomy for any condition); Patients with well-controlled type 1 diabetes can be enrolled.
6. With serious chronic diseases, such as serious cardiovascular diseases, hypertension, diabetes, liver and kidney diseases, malignant tumors, and so on.
7. With severe neurological disease (epilepsy, convulsions or convulsions) or mental illness.
8. With doctor-diagnosed abnormal blood coagulation function (e.g., lack of blood coagulation factors, hemorrhagic disorders, platelet abnormality) or obvious bruising or blood coagulation.
9. Had immunosuppressive therapy, cytotoxic therapy, inhaled corticosteroids (excluding allergic rhinitis corticosteroid spray therapy, superficial corticosteroid therapy for acute noncomplicated dermatitis) in the past 6 months.
10. Long-term history of alcohol or drug abuse.
11. Received blood products within 3 months prior to receiving the investigational vaccine, or planning to receive the above treatments during the study period.
12. Received other investigational vaccine or drugs in the past 30 days.
13. Received live attenuated vaccines in the past 14 days.
14. Received inactivated or subunit vaccines in the past 7 days.
15. With onset of various acute or chronic diseases within 7 days prior to the study.
16. With fever on the day of scheduled vaccination, axillary temperature was >37.0℃ before vaccination.
17. Women who are breastfeeding, pregnant, or planning to become pregnant during the study period (based on participants' self-reports and urine pregnancy test results).
18. Participating in or planning to participate in clinical trials of other vaccines or drugs.
19. Has any other factors that are not suitable for participating in the clinical trial according to the investigators’ judgment.

**2. Immunogenicity Testing Method**

Serum samples for all participants were taken at baseline and 28 days after the booster dose, and for a subset of participants, additional serum samples were collected at 7 and 14 days safter vaccination. Back-up serum samples at 28 days after two initial doses of vaccination were also collected from the previous lot-to-lot consistency study on CoronaVac. Neutralizing antibodies against SARS-CoV-2 ancestral strain (SARS-CoV-2/human/CHN/CN1/2020, GenBank accession number MT407649.1), Delta, and Omicron BA.1 and BA.5 were quantified using a micro cytopathogenic effect assay. Several measures were taken to control the quality of the micro cytopathogenic effect assay, including virus back-titration for each batch of tests to determine whether the amount of virus was within the range of 32-320 tissue culture infectious dose (TCID_50_) per 50 μL. Two types of positive antibody control, a negative antibody control, a serum toxicity control, and a cell control were included for each test. Detection was done by the National Institute for Food and Drug Control.

**Detection Method of Neutralization Potency against live SARS-CoV-2 ancestral strain, Delta and Omicron BA.1 and BA.5**

Micro cytopathic effect assay was adopted.

**Serum treatment:** all serum samples were inactivated at 56°C in a water bath for 30 minutes. **Medium addition:** the cell maintenance medium was added to the cell control group at 100 μL/well, and 50 μL/well of maintenance medium was supplemented to the to-be-tested serum group, virus back titration group and positive control group from the second dilution.

**Dilution of the serum sample:** The serum was diluted four-fold (60 μL sample + 180 μL maintenance medium) with cell maintenance medium (2% newborn calf serum199 (2% sodium hydrogen carbonate) cell maintenance medium). The diluted serum was added to the cell plate at 100 μL/well, and each sample was diluted to 2 wells in parallel. 50 μL of the mixture in the first dilution was pipetted into the next dilution, and the mixture was pipetted up and down for 8-10 times. The mixture was diluted to the appropriate dilution range by this method, and 50 μL of the last dilution was discarded, and 50 μL of the diluted sample was retained in each well.

**Dilution of the virus for neutralization:** the SAR-CoV-2 used for neutralization was diluted to 100CCID50/0.05ml by titer.

**Neutralization:** Serum of different dilutions was mixed with 100CCID50/0.05ml virus liquid in equal volume (50 μL+50 μL), respectively, and then incubated in an incubator at 36.5°C, 5%CO2 for 2h.

**Experimental control:** Negative serum control, positive serum control, serum sample and cell control were set simultaneously.

**Virus Back Titration:** The virus suspension diluted to 100 CCID50/0.05 mL was diluted via ten-fold serial dilution, i.e. diluted to 10 CCID50/0.05 mL, 1 CCID50/0.05 mL and 0.1 CCID50/0.05 mL, and added to the 96-well cell plate respectively, 12 well per dilution and 50 μL per well, then 50 μL of cell maintenance medium was added to each well, and the plate was incubated in an incubator at 36.5°C, 5% CO_2_ for 5 days.

**Cell Inoculation and Culture:** After incubation, 100μL of Vero cell suspension (cell concentration: 1.0-2.0×105 cell/mL) was added to each well, and then incubated in an incubator at 36.5°C, 5% CO_2_ for 5 days.

**Interpretation of the Results:** It was observed for the cytopathic effect after cultured for 3-5 days, and the neutralizing antibody titer of the to-be-tested serum sample was determined according to the observation results of the cytopathic effect (CPE) on the 5^th^ day. The reciprocal of the highest serum dilution without cytopathic effects the end titer. When 1 of the 2 wells of the highest dilution serum shows CPE, while the other does not, the reciprocal of the dilution should be the neutralizing antibody titer of the serum specimen; the reciprocal of the mean dilution of the two wells should be the neutralizing antibody titer of the serum specimen when the 2 wells with the highest dilution are completely pathological while the adjacent 2 wells with low dilution are not pathological completely; when 1 of two adjacent wells is pathological while the other not, the reciprocal of the average dilutions of 2 wells should be the neutralizing antibody titer of the serum specimen. For example, 2 wells with high dilution of 1:8 have a complete CPE, while the adjacent 2 wells with low dilution of 1:16 have no CPE; or in 2 adjacent wells with dilutions of 1:8 and 1:16, one has a CPE, while the other does not. In this case, the reciprocal 12 of the average dilutions of 2 wells is the neutralizing antibody titer of the serum.

**3. Hypotheses:**

Hypotheses were set separately for the 2C and 3C cohorts: For the primary objectives on immunogenicity, there are 3 hypotheses to be tested, and the trial would be considered to meet its primary objectives for each cohort if all hypotheses are met:

1. A booster dose of Omicron vaccine in 2C or 3C cohort is superior to a booster dose of CoronaVac in the 3C cohort, based on the GMT ratio of Omicron vaccine as compared with CoronaVac against Omicron BA.1 at 28 days with a superiority margin of 1.
2. A booster dose of Omicron vaccine in 2C or 3C cohort is superior to a booster dose of CoronaVac in the 3C cohort, based on the seroconversion rate difference of Omicron vaccine as compared with CoronaVac against Omicron BA.1 at 28 days with a superiority margin of 0%.
3. A booster dose of Omicron vaccine in 2C or 3C cohort is non-inferior to the two initial doses of CoronaVac in the previous clinical trial, based on the GMT ratio of Omicron vaccine against Omicron BA.1 as compared with CoronaVac against ancestral strain at 28 days with a non-inferiority margin of 2/3.

**Table S1. Baseline demographic and clinical characteristics of the participants in different ages (18-59 years and ≥60 years).**

|  | **2C cohort** | | **3C cohort** | | **P value*** | **Historical cohort** |
| --- | --- | --- | --- | --- | --- | --- |
| **Characteristic** | **Omicron vaccine booster** | **CoronaVac booster** | **Omicron vaccine booster** | **CoronaVac booster** |  |  |
| **18-59 years, N** | **399** | **203** | **396** | **199** |  | **250** |
| Age, years |  |  |  |  |  |  |
| Mean (SD) | 37.9 (10.0) | 37.2 (10.1) | 44.7 (9.8) | 45.1 (9.9) | <0.0001 | 34.8 (5.4) |
| Median (range) | 36 (18, 59) | 34 (18, 59) | 46 (21, 59) | 46 (20, 59) |  | 34 (26, 45) |
| Male, n (%) | 246 (61.65) | 137 (67.49) | 179 (45.20) | 97 (48.74) | 0.0026 | 118 (47.20) |
| Ethnic, n (%) |  |  |  |  |  |  |
| Han | 399 (100.00) | 203 (100.00) | 395 (99.75) | 199 (100.00) | - | 249 (99.60) |
| Hui | 0 (0.00) | 0 (0.00) | 1 (0.25) | 0 (0.00) |  | 1 (0.40) |
| Height, m |  |  |  |  |  |  |
| Mean (SD) | 1.67 (0.09) | 1.69 (0.08) | 1.64 (0.08) | 1.64 (0.08) | 0.0001 | 1.66 (0.08) |
| Weight, kg |  |  |  |  |  |  |
| Mean (SD) | 71.6 (14.5) | 75.6 (15.9) | 68.8 (12.4) | 70.0 (13.5) | 0.1894 | 71.5 (14.8) |
| Time to the latest dose, days |  |  |  |  |  |  |
| Mean (SD) | 339.8 (34.2) | 343.3 (30.1) | 205.9 (14.9) | 205.3 (14.8) | <0.0001 | NA |
| Median (range) | 349 (183, 407) | 355 (208, 402) | 206 (167, 241) | 206 (181, 235) |  | NA |
| **≥60 years, N** | **99** | **48** | **103** | **50** |  | **0** |
| Age, years |  |  |  |  |  |  |
| Mean (SD) | 66.1 (4.2) | 65.9 (3.8) | 64.8 (3.9) | 64.4 (3.6) | 0.0214 | NA |
| Median (range) | 65 (60, 77) | 65 (60, 74) | 64 (60, 77) | 63 (60, 73) |  | NA |
| Male, n (%) | 55 (55.56) | 29 (60.42) | 52 (50.49) | 32 (64.00) | 0.3234 | NA |
| Ethnic, n (%) |  |  |  |  |  |  |
| Han | 99 (100.00) | 48 (100.00) | 103 (100.00) | 50 (100.00) | - | NA |
| Height, m |  |  |  |  |  |  |
| Mean (SD) | 1.62 (0.07) | 1.63 (0.08) | 1.63 (0.09) | 1.63 (0.09) | 0.5242 | NA |
| Weight, kg |  |  |  |  |  |  |
| Mean (SD) | 65.8 (9.5) | 66.3 (8.6) | 68.8 (12.5) | 68.6 (12.1) | 0.1628 | NA |
| Time to the latest dose, days |  |  |  |  |  |  |
| Mean (SD) | 332.4 (23.8) | 332.0 (24.4) | 193.0 (8.1) | 192.0 (7.2) | <0.0001 | NA |
| Median (range) | 332 (282, 401) | 333 (273, 390) | 191 (181, 221) | 190.5 (182, 221) |  | NA |

Note: Results are shown for the intention-to-treat population (FAS). Historical cohort is the selected back-up serum samples from the previous lot-to-lot clinical trial. *: P values were calculated for comparing the characteristic differences between the 2C trial group and the 3C control group.

**Table S2. Adverse reactions within 28 days after booster vaccination, by Grade.**

| **2C and 3C combined cohort** | **All participants** | | | **18-59 years** | | | **≥60 years** | | |
| --- | --- | --- | --- | --- | --- | --- | --- | --- | --- |
|  | **Omicron vaccine booster group (N=998)** | **CoronaVac booster group (N=501)** | **P value** | **Omicron vaccine booster group (N=796)** | **CoronaVac booster group (N=403)** | **P value** | **Omicron vaccine booster group (N=202)** | **CoronaVac booster group (N=98)** | **P value** |
|  | **n (%)** | **n (%)** |  | **n (%)** | **n (%)** |  | **n (%)** | **n (%)** |  |
| **Total AR** | 114 (11.42) | 69 (13.77) | 0.2096 | 99 (12.44) | 53 (13.15) | 0.7143 | 15 (7.43) | 16 (16.33) | 0.025 |
| Grade 1 | 107 (10.72) | 69 (13.77) | 0.0891 | 94 (11.81) | 53 (13.15) | 0.5148 | 13 (6.44) | 16 (16.33) | 0.0112 |
| Grade 2 | 9 (0.90) | 2 (0.40) | 0.3538 | 7 (0.88) | 1 (0.25) | 0.2798 | 2 (0.99) | 1 (1.02) | 1 |
| Grade 3 | 2 (0.20) | 0 (0.00) | 0.5547 | 2 (0.25) | 0 (0.00) | 0.5533 | 0 (0.00) | 0 (0.00) | 1 |
| **Solicited AR** | 111 (11.12) | 69 (13.77) | 0.152 | 96 (12.06) | 53 (13.15) | 0.5797 | 15 (7.43) | 16 (16.33) | 0.025 |
| Grade 1 | 105 (10.52) | 69 (13.77) | 0.0725 | 92 (11.56) | 53 (13.15) | 0.4534 | 13 (6.44) | 16 (16.33) | 0.0112 |
| Grade 2 | 8 (0.80) | 1 (0.20) | 0.2866 | 6 (0.75) | 0 (0.00) | 0.1874 | 2 (0.99) | 1 (1.02) | 1 |
| Grade 3 | 2 (0.20) | 0 (0.00) | 0.5547 | 2 (0.25) | 0 (0.00) | 0.5533 | 0 (0.00) | 0 (0.00) | 1 |
| **Local AR** | 67 (6.71) | 47 (9.38) | 0.0787 | 55 (6.91) | 37 (9.18) | 0.1692 | 12 (5.94) | 10 (10.20) | 0.2368 |
| Grade 1 | 63 (6.31) | 47 (9.38) | 0.0357 | 52 (6.53) | 37 (9.18) | 0.1034 | 11 (5.45) | 10 (10.20) | 0.1499 |
| Grade 2 | 6 (0.60) | 1 (0.20) | 0.4353 | 5 (0.63) | 0 (0.00) | 0.1747 | 1 (0.50) | 1 (1.02) | 0.5474 |
| Grade 3 | 1 (0.10) | 0 (0.00) | 1 | 1 (0.13) | 0 (0.00) | 1 | 0 (0.00) | 0 (0.00) | 1 |
| **Pain** | 56 (5.61) | 40 (7.98) | 0.0929 | 49 (6.16) | 32 (7.94) | 0.273 | 7 (3.47) | 8 (8.16) | 0.0937 |
| Grade 1 | 56 (5.61) | 40 (7.98) | 0.0929 | 49 (6.16) | 32 (7.94) | 0.273 | 7 (3.47) | 8 (8.16) | 0.0937 |
| **Induration** | 12 (1.20) | 13 (2.59) | 0.055 | 12 (1.51) | 11 (2.73) | 0.1802 | 0 (0.00) | 2 (2.04) | 0.106 |
| Grade 1 | 9 (0.90) | 12 (2.40) | 0.033 | 9 (1.13) | 11 (2.73) | 0.0546 | 0 (0.00) | 1 (1.02) | 0.3267 |
| Grade 2 | 3 (0.30) | 1 (0.20) | 1 | 3 (0.38) | 0 (0.00) | 0.555 | 0 (0.00) | 1 (1.02) | 0.3267 |
| **Pruritus** | 10 (1.00) | 12 (2.40) | 0.0411 | 7 (0.88) | 9 (2.23) | 0.0639 | 3 (1.49) | 3 (3.06) | 0.3962 |
| Grade 1 | 10 (1.00) | 12 (2.40) | 0.0411 | 7 (0.88) | 9 (2.23) | 0.0639 | 3 (1.49) | 3 (3.06) | 0.3962 |
| **Erythema** | 7 (0.70) | 6 (1.20) | 0.3789 | 6 (0.75) | 5 (1.24) | 0.5223 | 1 (0.50) | 1 (1.02) | 0.5474 |
| Grade 1 | 6 (0.60) | 6 (1.20) | 0.2312 | 5 (0.63) | 5 (1.24) | 0.3179 | 1 (0.50) | 1 (1.02) | 0.5474 |
| Grade 2 | 0 (0.00) | 0 (0.00) | 1 | 0 (0.00) | 0 (0.00) | 1 | 0 (0.00) | 0 (0.00) | 1 |
| Grade 3 | 1 (0.10) | 0 (0.00) | 1 | 1 (0.13) | 0 (0.00) | 1 | 0 (0.00) | 0 (0.00) | 1 |
| **Swelling** | 5 (0.50) | 8 (1.60) | 0.0395 | 4 (0.50) | 7 (1.74) | 0.0505 | 1 (0.50) | 1 (1.02) | 0.5474 |
| Grade 1 | 1 (0.10) | 7 (1.40) | 0.0026 | 1 (0.13) | 7 (1.74) | 0.0027 | 0 (0.00) | 0 (0.00) | 1 |
| Grade 2 | 4 (0.40) | 1 (0.20) | 0.6698 | 3 (0.38) | 0 (0.00) | 0.555 | 1 (0.50) | 1 (1.02) | 0.5474 |
| **Rash** | 2 (0.20) | 0 (0.00) | 0.5547 | 2 (0.25) | 0 (0.00) | 0.5533 | 0 (0.00) | 0 (0.00) | 1 |
| Grade 1 | 2 (0.20) | 0 (0.00) | 0.5547 | 2 (0.25) | 0 (0.00) | 0.5533 | 0 (0.00) | 0 (0.00) | 1 |
| **Systemic AR** | 58 (5.81) | 29 (5.79) | 1 | 55 (6.91) | 20 (4.96) | 0.2082 | 3 (1.49) | 9 (9.18) | 0.0027 |
| Grade 1 | 55 (5.51) | 29 (5.79) | 0.8129 | 53 (6.66) | 20 (4.96) | 0.3062 | 2 (0.99) | 9 (9.18) | 0.0009 |
| Grade 2 | 2 (0.20) | 0 (0.00) | 0.5547 | 1 (0.13) | 0 (0.00) | 1 | 1 (0.50) | 0 (0.00) | 1 |
| Grade 3 | 1 (0.10) | 0 (0.00) | 1 | 1 (0.13) | 0 (0.00) | 1 | 0 (0.00) | 0 (0.00) | 1 |
| **Fever** | 35 (3.51) | 22 (4.39) | 0.3939 | 33 (4.15) | 16 (3.97) | 1 | 2 (0.99) | 6 (6.12) | 0.0164 |
| Grade 1 | 33 (3.31) | 22 (4.39) | 0.3093 | 32 (4.02) | 16 (3.97) | 1 | 1 (0.50) | 6 (6.12) | 0.0056 |
| Grade 2 | 1 (0.10) | 0 (0.00) | 1 | 0 (0.00) | 0 (0.00) | 1 | 1 (0.50) | 0 (0.00) | 1 |
| Grade 3 | 1 (0.10) | 0 (0.00) | 1 | 1 (0.13) | 0 (0.00) | 1 | 0 (0.00) | 0 (0.00) | 1 |
| **Diarrhea** | 10 (1.00) | 3 (0.60) | 0.5617 | 10 (1.26) | 1 (0.25) | 0.1113 | 0 (0.00) | 2 (2.04) | 0.106 |
| Grade 1 | 10 (1.00) | 3 (0.60) | 0.5617 | 10 (1.26) | 1 (0.25) | 0.1113 | 0 (0.00) | 2 (2.04) | 0.106 |
| **Fatigue** | 9 (0.90) | 4 (0.80) | 1 | 8 (1.01) | 2 (0.50) | 0.5097 | 1 (0.50) | 2 (2.04) | 0.2496 |
| Grade 1 | 9 (0.90) | 4 (0.80) | 1 | 8 (1.01) | 2 (0.50) | 0.5097 | 1 (0.50) | 2 (2.04) | 0.2496 |
| **Headache** | 6 (0.60) | 6 (1.20) | 0.2312 | 6 (0.75) | 4 (0.99) | 0.74 | 0 (0.00) | 2 (2.04) | 0.106 |
| Grade 1 | 6 (0.60) | 6 (1.20) | 0.2312 | 6 (0.75) | 4 (0.99) | 0.74 | 0 (0.00) | 2 (2.04) | 0.106 |
| **Myalgia** | 4 (0.40) | 1 (0.20) | 0.6698 | 3 (0.38) | 1 (0.25) | 1 | 1 (0.50) | 0 (0.00) | 1 |
| Grade 1 | 4 (0.40) | 1 (0.20) | 0.6698 | 3 (0.38) | 1 (0.25) | 1 | 1 (0.50) | 0 (0.00) | 1 |
| **Cough** | 3 (0.30) | 1 (0.20) | 1 | 2 (0.25) | 0 (0.00) | 0.5533 | 1 (0.50) | 1 (1.02) | 0.5474 |
| Grade 1 | 2 (0.20) | 1 (0.20) | 1 | 1 (0.13) | 0 (0.00) | 1 | 1 (0.50) | 1 (1.02) | 0.5474 |
| Grade 2 | 1 (0.10) | 0 (0.00) | 1 | 1 (0.13) | 0 (0.00) | 1 | 0 (0.00) | 0 (0.00) | 1 |
| **Acute hypersensitive reaction** | 2 (0.20) | 0 (0.00) | 0.5547 | 2 (0.25) | 0 (0.00) | 0.5533 | 0 (0.00) | 0 (0.00) | 1 |
| Grade 1 | 2 (0.20) | 0 (0.00) | 0.5547 | 2 (0.25) | 0 (0.00) | 0.5533 | 0 (0.00) | 0 (0.00) | 1 |
| **Nausea** | 1 (0.10) | 0 (0.00) | 1 | 1 (0.13) | 0 (0.00) | 1 | 0 (0.00) | 0 (0.00) | 1 |
| Grade 1 | 1 (0.10) | 0 (0.00) | 1 | 1 (0.13) | 0 (0.00) | 1 | 0 (0.00) | 0 (0.00) | 1 |
| **Unsolicited AR** | 3 (0.30) | 1 (0.20) | 1 | 3 (0.38) | 1 (0.25) | 1 | 0 (0.00) | 0 (0.00) | 1 |
| Grade 1 | 2 (0.20) | 0 (0.00) | 0.5547 | 2 (0.25) | 0 (0.00) | 0.5533 | 0 (0.00) | 0 (0.00) | 1 |
| Grade 2 | 1 (0.10) | 1 (0.20) | 1 | 1 (0.13) | 1 (0.25) | 1 | 0 (0.00) | 0 (0.00) | 1 |
| **Oropharynx pain** | 1 (0.10) | 0 (0.00) | 1 | 1 (0.13) | 0 (0.00) | 1 | 0 (0.00) | 0 (0.00) | 1 |
| Grade 1 | 0 (0.00) | 0 (0.00) | 1 | 0 (0.00) | 0 (0.00) | 1 | 0 (0.00) | 0 (0.00) | 1 |
| Grade 2 | 1 (0.10) | 0 (0.00) | 1 | 1 (0.13) | 0 (0.00) | 1 | 0 (0.00) | 0 (0.00) | 1 |
| **Constipation** | 1 (0.10) | 0 (0.00) | 1 | 1 (0.13) | 0 (0.00) | 1 | 0 (0.00) | 0 (0.00) | 1 |
| Grade 1 | 1 (0.10) | 0 (0.00) | 1 | 1 (0.13) | 0 (0.00) | 1 | 0 (0.00) | 0 (0.00) | 1 |
| **Dizzy** | 1 (0.10) | 0 (0.00) | 1 | 1 (0.13) | 0 (0.00) | 1 | 0 (0.00) | 0 (0.00) | 1 |
| Grade 1 | 1 (0.10) | 0 (0.00) | 1 | 1 (0.13) | 0 (0.00) | 1 | 0 (0.00) | 0 (0.00) | 1 |
| **Vesicular exanthema** | 0 (0.00) | 1 (0.20) | 0.3342 | 0 (0.00) | 1 (0.25) | 0.3361 | 0 (0.00) | 0 (0.00) | 1 |
| Grade 1 | 0 (0.00) | 0 (0.00) | 1 | 0 (0.00) | 0 (0.00) | 1 | 0 (0.00) | 0 (0.00) | 1 |
| Grade 2 | 0 (0.00) | 1 (0.20) | 0.3342 | 0 (0.00) | 1 (0.25) | 0.3361 | 0 (0.00) | 0 (0.00) | 1 |

Note: Participants in the safety set of combined 2C and 3C cohort (998 participants in the Omicron vaccine group and 501 participants in the CoronaVac group). P values were calculated by Fisher’s exact test.

**Table S3. Immunogenicity subgroup analysis on day 28 after booster doses of Omicron vaccine and CoronaVac, as well as after two initial doses of CoronaVac, by age (18-59 years and ≥60 years).**

| **Variable** | **Omicron vaccine booster** | | **CoronaVac booster** | **Historical control group** |
| --- | --- | --- | --- | --- |
|  | **2C cohort** | **3C cohort** | **3C cohort** |  |
| **18-59 years, N** | **351** | **385** | **191** | **238** |
| **Against SARS-CoV-2** | **Omicron BA.1** | **Omicron BA.1** | **Omicron BA.1** | **ancestral strain** |
| **Day 0** |  |  |  |  |
| Observed GMT (95%CI) | 2.03 (2.00, 2.06) | 2.67 (2.53, 2.82) | 2.74 (2.51, 3.00) | / |
| **Day 28** |  |  |  |  |
| Observed GMT (95%CI) | 10.32 (9.28, 11.48) | 11.09 (9.95, 12.37) | 6.99 (6.06, 8.07) | 45.97 (41.08, 51.44) |
| GMI (95%CI) | 5.09 (4.57, 5.66) | 4.16 (3.77, 4.58) | 2.55 (2.25, 2.88) | / |
| Seropositive rate (95%CI) | 62.11% (56.81, 67.20) | 66.23% (61.27, 70.95) | 46.60% (39.36, 53.94) | 97.48% (94.59, 99.07) |
| **Superiority test** |  |  |  |  |
| Estimated GMT (95%CI) | 11.25 (10.15, 12.48) | 11.18 (10.19, 12.27) | 5.96 (5.17, 6.88)# 6.88 (6.03, 7.85)¶ | / |
| **GMT ratio (95%CI)** | **1.89 (1.57, 2.26)** | **1.63 (1.38, 1.91)** | Ref | / |
| Seroconversion rate (95%CI) | 38.75% (33.62, 44.06) | 35.06% (30.30, 40.06) | 17.28% (12.20, 23.40) | / |
| **Seroconversion rate differences (95%CI)** | **21.47% (13.76, 28.60)** | **17.79% (10.29, 24.69)** | Ref | / |
| **Non-inferiority test** |  |  |  |  |
| Estimated GMT (95%CI) | 10.32 (9.33, 11.41) | 11.09 (10.03, 12.28) | / | 45.97(40.67, 51.96)# 45.97(40.41, 52.29)¶ |
| **GMT ratio (95%CI)** | **0.22 (0.19, 0.26)** | **0.24 (0.20, 0.28)** | / | Ref |
| **≥60 years, N** | **86** | **96** | **47** | **0** |
| **Against SARS-CoV-2** | **Omicron BA.1** | **Omicron BA.1** | **Omicron BA.1** | **ancestral strain** |
| **Day 0** |  |  |  |  |
| Observed GMT (95%CI) | 2.00 (2.00, 2.00) | 2.56 (2.28, 2.87) | 2.71 (2.21, 3.32) | / |
| **Day 28** |  |  |  |  |
| Observed GMT (95%CI) | 6.08 (4.96, 7.46) | 7.89 (6.25, 9.96) | 4.92 (3.63, 6.66) | / |
| GMI (95%CI) | 3.04 (2.48, 3.73) | 3.08 (2.54, 3.75) | 1.82 (1.45, 2.28) | / |
| Seropositive rate (95%CI) | 46.51% (35.68, 57.59) | 54.17% (43.69, 64.38) | 36.17% (22.67, 51.48) | / |
| **Superiority test** |  |  |  |  |
| Estimated GMT (95%CI) | 6.76 (5.56, 8.22) | 8.05 (6.69, 9.67) | 4.06 (3.10, 5.31)# 4.73 (3.63, 6.14)¶ | / |
| **GMT ratio (95%CI)** | **1.67 (1.18, 2.34)** | **1.70 (1.24, 2.35)** | Ref | / |
| Seroconversion rate (95%CI) | 19.77% (11.96, 29.75) | 28.13% (19.42, 38.22) | 10.64% (3.55, 23.10) | / |
| **Seroconversion rate differences (95%CI)** | **9.13% (-4.72, 20.92)** | **17.49% (3.26, 29.42)** | Ref | / |

Note: Results are shown for the participants in PPS3 (the primary analysis dataset). The seroconversion refers to the neutralizing antibody titer < LLOQ (1:4) before vaccination but ≥4-fold LLOQ (1:16) after vaccination, or neutralizing antibody ≥1:4 before vaccination but ≥4 times increase after vaccination. The positive cutoff value of the neutralizing antibody titer was 1:8. #: Estimated GMT value when comparing with Omicron vaccine group in 2C cohort. ¶: Estimated GMT value when comparing with Omicron vaccine group in 3C cohort.

**Figure S1. Observed neutralizing antibody levels against Delta and ancestral strain****s before and 28 days after booster doses of Omicron vaccine or CoronaVac in 2C cohort (A) and 3C cohort (B), by age (all, 18-59 years, and ≥60 years).**

**
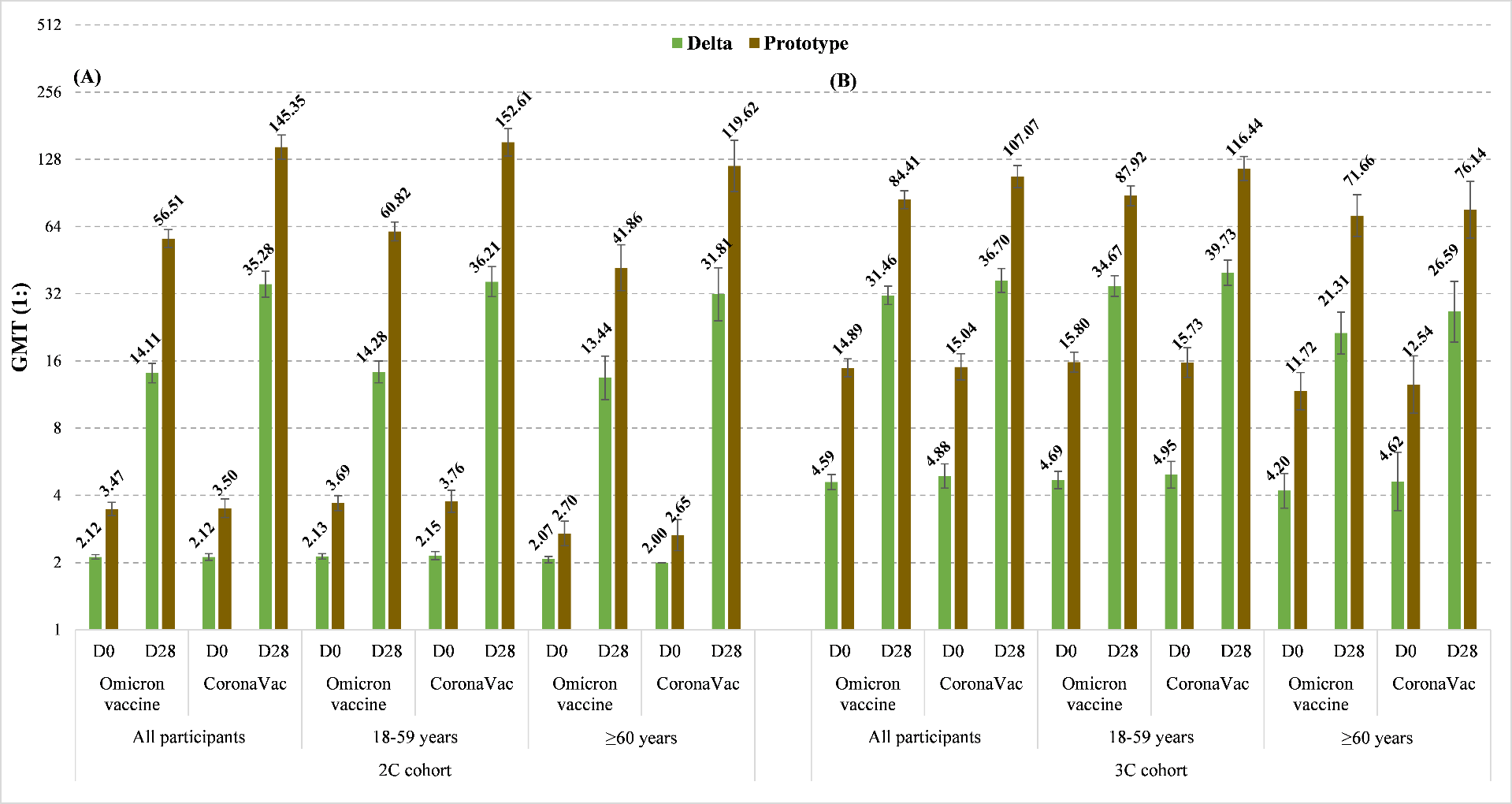
**

Note: Results are shown for the participants in PPS3. Neutralizing antibody titers lower than LLOQ (1:4) are presented as half of LLOQ. The GMTs are shown on the top of histograms and their 95%CIs are indicated by the error bars.

**Figure S2. Observed neutralizing antibody levels against Omicron BA.1, BA.5, Delta and ancestral strains before and 7, 14 and 28 days after booster doses of Omicron vaccine or CoronaVac in 2C cohort (A) and 3C cohort (B) in all age participants.**


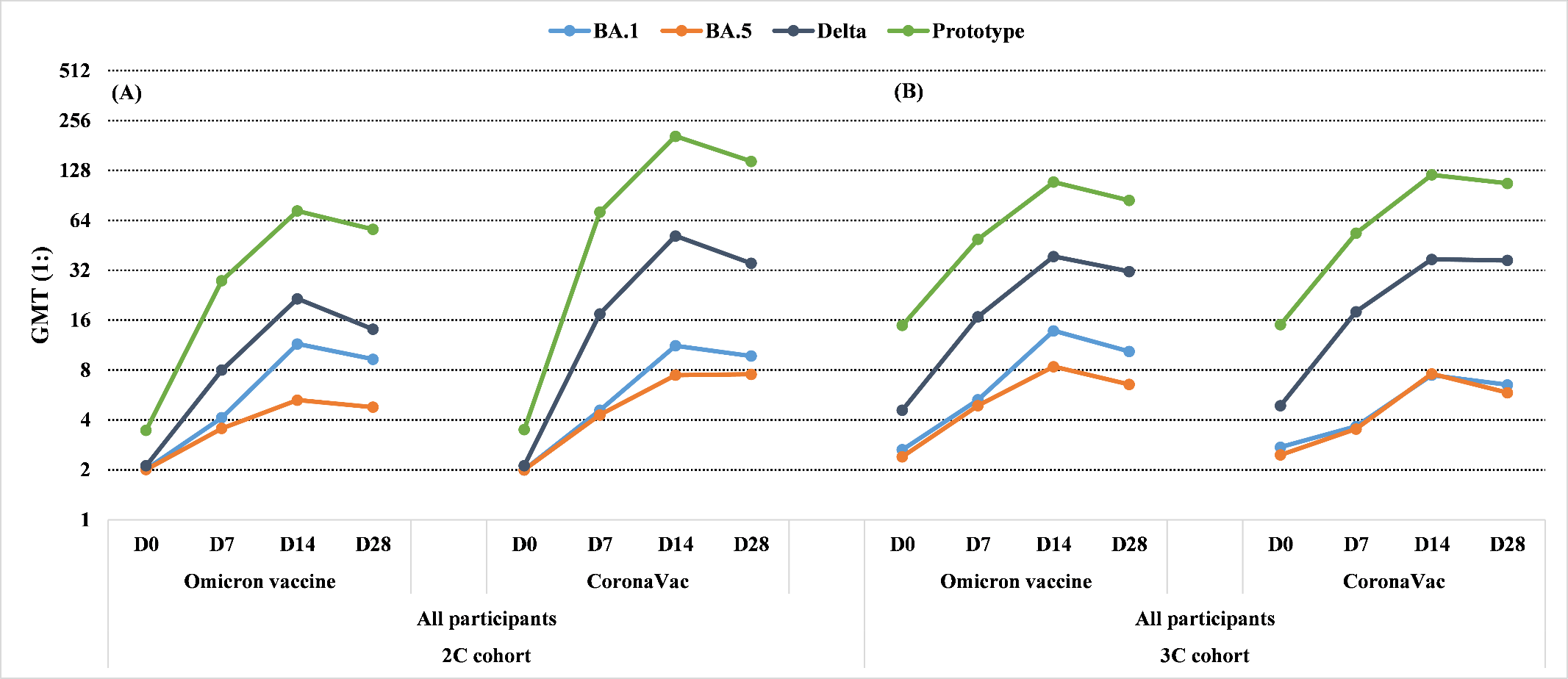


Note: Results are shown for the participants in PPS1, PPS2 and PPS3. The neutralizing antibody levels at 7 and 14 days were measured in a subset of participants. The number of participants at different timepoints was shown in Table S5. Neutralizing antibody titers lower than LLOQ (1:4) are presented as half of LLOQ.

**Table S4. Observed neutralizing antibody levels against Omicron BA.1, BA.5, Delta and ancestral strains before and 7, 14 and 28 days after booster doses of Omicron vaccine or CoronaVac in 2C and 3C cohorts, by age (all, 18-59 years, and ≥60 years)**

| **Variable** | **Omicron vaccine booster** | | | |  | **CoronaVac booster** | | | |
| --- | --- | --- | --- | --- | --- | --- | --- | --- | --- |
|  | **Day 0** | **Day 7** | **Day 14** | **Day 28** |  | **Day 0** | **Day 7** | **Day 14** | **Day 28** |
| **2C cohort, GMT (95%CI)** |  |  |  |  |  |  |  |  |  |
| **All participants, N** | **437** | **78** | **78** | **437** |  | **220** | **36** | **36** | **220** |
| BA.1 | 2.02 (2.00, 2.05) | 4.13 (3.43, 4.96) | 11.51 (8.92, 14.84) | 9.30 (8.45, 10.24) |  | 2.00 (2.00, 2.00) | 4.58 (3.38, 6.19) | 11.23 (7.87, 16.03) | 9.73 (8.54, 11.09) |
| BA.5 | 2.01 (2.00, 2.02) | 3.56 (3.05, 4.14) | 5.27 (4.22, 6.58) | 4.78 (4.41, 5.19) |  | 2.00 (2.00, 2.00) | 4.29 (3.38, 5.43) | 7.46 (5.28, 10.54) | 7.55 (6.63, 8.60) |
| Delta | 2.12 (2.07, 2.17) | 8.00 (6.27, 10.22) | 21.56 (16.70, 27.83) | 14.11 (12.78, 15.59) |  | 2.12 (2.05, 2.19) | 17.48 (11.47, 26.65) | 51.56 (38.03, 69.90) | 35.28 (30.85, 40.35) |
| Prototype | 3.47 (3.24, 3.72) | 27.75 (21.29, 36.18) | 72.94 (58.25, 91.35) | 56.51 (51.57, 61.92) |  | 3.50 (3.18, 3.86) | 71.76 (50.29, 102.40) | 205.94 (153.25, 276.75) | 145.35 (128.34, 164.62) |
| **18-59 years, N** | **351** | **41** | **41** | **351** |  | **176** | **18** | **19** | **176** |
| BA.1 | 2.03 (2.00, 2.06) | 4.45 (3.41, 5.80) | 16.42 (11.49, 23.47) | 10.32 (9.28, 11.48) |  | 2.00 (2.00, 2.00) | 3.86 (2.48, 6.01) | 13.84 (7.61, 25.15) | 10.73 (9.24, 12.46) |
| BA.5 | 2.01 (2.00, 2.03) | 3.11 (2.54, 3.81) | 5.64 (4.01, 7.94) | 4.93 (4.50, 5.41) |  | 2.00 (2.00, 2.00) | 3.03 (2.24, 4.10) | 9.49 (5.46, 16.49) | 8.04 (6.92, 9.34) |
| Delta | 2.13 (2.07, 2.19) | 6.82 (4.89, 9.51) | 23.69 (16.88, 33.24) | 14.28 (12.78, 15.96) |  | 2.15 (2.06, 2.24) | 12.18 (6.59, 22.53) | 59.70 (37.30, 95.56) | 36.21 (31.03, 42.25) |
| Prototype | 3.69 (3.42, 3.99) | 28.93 (19.37, 43.22) | 90.82 (68.27, 120.80) | 60.82 (55.19, 67.04) |  | 3.76 (3.36, 4.21) | 65.08 (37.36, 113.35) | 268.09 (178.53, 402.57) | 152.61 (132.53, 175.73) |
| **≥60 years, N** | **86** | **37** | **37** | **86** |  | **44** | **18** | **17** | **44** |
| BA.1 | 2.00 (2.00, 2.00) | 3.80 (2.92, 4.95) | 7.76 (5.56, 10.82) | 6.08 (4.96, 7.46) |  | 2.00 (2.00, 2.00) | 5.43 (3.49, 8.44) | 8.90 (6.02, 13.14) | 6.57 (5.20, 8.31) |
| BA.5 | 2.00 (2.00, 2.00) | 4.13 (3.28, 5.20) | 4.88 (3.65, 6.52) | 4.22 (3.55, 5.02) |  | 2.00 (2.00, 2.00) | 6.07 (4.47, 8.25) | 5.70 (3.75, 8.67) | 5.89 (4.60, 7.55) |
| Delta | 2.07 (2.00, 2.13) | 9.56 (6.63, 13.80) | 19.43 (13.02, 28.99) | 13.44 (10.75, 16.81) |  | 2.00 (2.00, 2.00) | 25.09 (13.96, 45.09) | 43.77 (28.97, 66.11) | 31.81 (24.22, 41.78) |
| Prototype | 2.70 (2.38, 3.06) | 26.51 (18.52, 37.94) | 57.22 (40.29, 81.26) | 41.86 (33.07, 52.99) |  | 2.65 (2.26, 3.11) | 79.13 (48.02, 130.38) | 153.37 (99.95, 235.34) | 119.62 (91.76, 155.92) |
| **3C cohort, GMT (95%CI)** |  |  |  |  |  |  |  |  |  |
| **All participants, N** | **481** | **76** | **77** | **481** |  | **238** | **38** | **38** | **238** |
| BA.1 | 2.65 (2.52, 2.78) | 5.30 (4.16, 6.75) | 13.82 (10.53, 18.13) | 10.36 (9.39, 11.44) |  | 2.74 (2.52, 2.97) | 3.66 (2.80, 4.77) | 7.46 (5.34, 10.43) | 6.52 (5.73, 7.43) |
| BA.5 | 2.40 (2.31, 2.50) | 4.89 (3.91, 6.13) | 8.40 (6.51, 10.83) | 6.56 (6.00, 7.17) |  | 2.46 (2.31, 2.62) | 3.53 (2.85, 4.37) | 7.60 (5.45, 10.59) | 5.85 (5.18, 6.60) |
| Delta | 4.59 (4.24, 4.97) | 16.79 (12.88, 21.89) | 38.80 (29.00, 51.91) | 31.46 (28.58, 34.63) |  | 4.88 (4.31, 5.53) | 18.00 (12.07, 26.83) | 37.27 (26.10, 53.22) | 36.70 (32.46, 41.51) |
| Prototype | 14.89 (13.58, 16.32) | 49.27 (37.31, 65.06) | 109.00 (83.95, 141.53) | 84.41 (76.95, 92.59) |  | 15.04 (13.14, 17.23) | 53.61 (36.48, 78.78) | 120.52 (87.71, 165.61) | 107.07 (95.31, 120.27) |
| **18-59 years, N** | **385** | **38** | **39** | **358** |  | **191** | **20** | **20** | **191** |
| BA.1 | 2.67 (2.53, 2.82) | 7.20 (5.02, 10.31) | 14.87 (10.15, 21.77) | 11.09 (9.95, 12.37) |  | 2.74 (2.51, 3.00) | 3.88 (2.64, 5.70) | 7.75 (5.02, 11.98) | 6.99 (6.06, 8.07) |
| BA.5 | 2.44 (2.33, 2.56) | 5.96 (4.20, 8.45) | 8.77 (6.20, 12.41) | 6.86 (6.19, 7.59) |  | 2.44 (2.27, 2.61) | 3.40 (2.52, 4.60) | 7.28 (4.79, 11.05) | 5.92 (5.19, 6.76) |
| Delta | 4.69 (4.29, 5.13) | 21.24 (14.38, 31.37) | 48.26 (31.42, 74.12) | 34.67 (31.19, 38.53) |  | 4.95 (4.31, 5.68) | 20.70 (11.63, 36.85) | 44.76 (27.11, 73.89) | 39.73 (34.84, 45.31) |
| Prototype | 15.80 (14.24, 17.53) | 57.41 (37.24, 88.50) | 116.84 (79.14, 172.52) | 87.92 (79.36, 97.40) |  | 15.73 (13.50, 18.34) | 57.72 (32.20, 103.45) | 131.83 (88.58, 196.18) | 116.44 (102.82, 131.86) |
| **≥60 years, N** | **96** | **38** | **38** | **96** |  | **47** | **18** | **18** | **47** |
| BA.1 | 2.56 (2.28, 2.87) | 3.90 (2.87, 5.32) | 12.82 (8.57, 19.20) | 7.89 (6.25, 9.96) |  | 2.71 (2.21, 3.32) | 3.43 (2.29, 5.13) | 7.15 (4.07, 12.58) | 4.92 (3.63, 6.66) |
| BA.5 | 2.25 (2.08, 2.43) | 4.02 (3.02, 5.35) | 8.03 (5.44, 11.84) | 5.49 (4.57, 6.61) |  | 2.57 (2.20, 2.99) | 3.67 (2.62, 5.14) | 7.98 (4.50, 14.12) | 5.55 (4.08, 7.55) |
| Delta | 4.20 (3.51, 5.01) | 13.28 (9.25, 19.05) | 31.01 (20.80, 46.24) | 21.31 (17.18, 26.42) |  | 4.62 (3.42, 6.24) | 15.41 (8.44, 28.13) | 30.41 (17.68, 52.32) | 26.59 (19.43, 36.39) |
| Prototype | 11.72 (9.68, 14.19) | 42.29 (29.43, 60.77) | 101.50 (70.55, 146.04) | 71.66 (57.75, 88.93) |  | 12.54 (9.32, 16.87) | 49.38 (28.42, 85.81) | 109.09 (63.15, 188.47) | 76.14 (56.86, 101.95) |

Note: Results are shown for the participants in PPS1, PPS2 and PPS3. The neutralizing antibody levels at 7 and 14 days were measured in a subset of participants, which were the first 80 enrolled participants in the Omicron vaccine group and the first 40 enrolled participants in the CoronaVac group.
